# Does Metastatic Lymph Node SUVmax Predict Survival in Patients with Esophageal Cancer?

**DOI:** 10.4274/mirt.36744

**Published:** 2015-11-02

**Authors:** Betül Vatankulu, Yasemin Şanlı, Esra Kaytan Sağlam, Serkan Kuyumcu, Zeynep Gözde Özkan, Ebru Yılmaz, Sevim Purisa, Işık Adalet

**Affiliations:** 1 Istanbul University Cerrahpasa Faculty of Medicine, Department of Nuclear Medicine, İstanbul, Turkey; 2 İstanbul University İstanbul Faculty of Medicine, Department of Nuclear Medicine, İstanbul, Turkey; 3 İstanbul University İstanbul Faculty of Medicine, Department of Radiation Oncology, İstanbul, Turkey; 4 İstanbul University İstanbul Faculty of Medicine, Department of Biostatistics, İstanbul, Turkey

**Keywords:** Esophageal cancer, FDG positron-emission tomography/computed tomography, Survival, Lymph node, SUVmax

## Abstract

**Objective:**

We aimed to investigate the SUVmax of primary tumor and metastatic lymph node in predicting survival in patients with esophageal cancer.

**Methods:**

We retrospectively analyzed patients with esophageal cancer between 2009 and 2011 who had FDG positron-emission tomography (PET)/computed tomography (CT). All patients were followed-up to 2013. Clinical staging, SUVmax of primary tumor and metastatic lymph node were evaluated.

**Results:**

One hundred seven patients were included in the study. All patients were followed-up between 2 and 49 months. The mean SUVmax of primary tumor and metastatic lymph node were 19.3±8.8 and 10.4±9.1, respectively. Metastatic lymph node SUVmax had an effect in predicting survival whereas primary tumor SUVmax did not have an effect (p=0.014 and p=0.262, respectively). Multivariate Cox regression analysis showed that clinical stage of the disease was the only independent factor predicting survival (p=0.001).

**Conclusion:**

Among patients with esophageal cancer, the value of primary tumor SUVmax did not have an effect on survival. Clinical stage assessed with FDG PET/CT imaging was found to predict survival in esophageal carcinoma. Additionally, lymph node SUVmax was identified as a new parameter in predicting survival in the present study.

## Abstract (tr)

**Amaç:**

Çalışmamızda özofagus kanserli hastalarda primer tümör SUDmax ve metastatik lenf nodu SUDmax’ın sağ kalım üzerine olan etkisini araştırmayı amaçladık.

**Yöntem:**

2009 ve 2011 yılları arasında evreleme amacıyla FDG pozitron emisyon tomografisi (PET)/bilgisayarlı tomografi (BT) yapılan özofagus kanseri tanılı hastalar retrospektif olarak incelendi. Tüm hastalar Eylül 2013 yılına kadar takip edildi. Histopatoloji, lokalizasyon, klinik evre, primer tümör ve metatastatik SUVmax kaydedildi. Tanımlanan parametreler arasındaki ilişkiler ve sağ kalım univaryant ve multivaryant Cox regresyon analizi ile değerlendirildi.

**Bulgular:**

Çalışmaya 107 hasta dahil edildi. Hastaların hepsi 2-49 ay arasında takip edildi. Primer tümör ve metastatik lenf nodu ortalama SUDmax sırasıyla 19,3±8,8 ve 10,4±9,1 olarak hesaplandı. Metastatik lenf nodu SUDmax sağ kalım üzerine etkili iken primer tümör SUDmax’ın sağ kalım üzerine etkisi olmadığı görüldü (p=0,014 ve p=0,262, sırasıyla). Klinik evrenin sağ kalımı belirlemede multivaryant Cox regresyon analizi ile sağ kalımı belirlemede bağımsız tek faktör olduğu görüldü (p=0,001).

**Sonuç:**

Özofagus kanseri tanılı hastalarda primer tümör SUDmax’ın sağ kalım üzerine etkisi olmadığı görüldü. FDG PET/CT ile belirlenen klinik evrenin sağ kalımı belirlediği görüldü. Ayrıca çalışmamızda metastatik lenf nodu SUDmax’ın sağ kalımı öngörmede yeni bir parametre olabileceği görülmektedir.

## INTRODUCTION

Esophageal cancer constitutes 1.5-2% of all cancers and 5-7% of those in the gastrointestinal tract. It is one of the most lethal of all cancers, and is known to be aggressive, showing significant progression in early stages via metastasis. Long-term survival is low, despite appropriate treatment. The assessment of survival in advance plays an important role in the treatment of the disease (1,2,3,4).

Although the significance of positron-emission tomography/computed tomography (PET/CT) imaging in clinical staging is well known, numerous studies have evaluated the effect of the maximum standard uptake value (SUVmax) obtained via PET/CT before surgery on survival. While some authors reported that primary tumor SUVmax is associated with survival, others indicated no such association (1,2,3,4,5,6,7,8,9). However, there are no clinical studies on the effect of metastatic lymph node SUVmax on survival in esophageal cancer.

The purpose of this retrospective study was to investigate the effect of primary tumor SUVmax, metastatic lymph node SUVmax, and clinical staging as determined by FDG PET/CT on survival of histopathologically proven esophageal cancer patients in our clinics, during a 2-49 month follow-up period.

## MATERIALS AND METHODS

Patients who were histopathologically diagnosed as esophageal cancer and referred to İstanbul University Faculty of Medicine Department of Nuclear Medicine PET/CT Unit for staging by FDG PET/CT imaging between May 2009 and December 2011 were retrospectively evaluated. Clinical follow-up was conducted until March 2013.

The patients were divided into two groups according to their histopathological diagnosis as patients with either squamous cell carcinoma or adenocarcinoma. Tumor localizations were categorized into five groups: cervical, upper thoracic, middle thoracic, lower thoracic, and middle + lower thoracic.

Clinical staging was performed using the tumor-node-metastasis (TNM) staging, a six-stage classification system provided by the American Joint Committee on Cancer (AJCC), after assessment of the results of CT images, endoscopy, pathology reports, and PET/CT imaging as a whole.

### Positron-Emission Tomography/Computed Tomography Protocol

#### Patient Preparation

Patients were instructed to fast for at least 6 h before imaging. The oral anti-diabetic metformine or its derivatives were discontinued in diabetic patients 3 days before the procedure, to prevent colonic uptake. Patients using insulin were allowed to take their long-acting insulin treatment 12 h before FDG injection. Six hours before imaging, patients were administered oral contrast material to delineate the intestine.

The blood glucose level of each patient was measured and then FDG was administered by establishing vascular access for those patients with blood glucose lower than 200 mg dL−1 to prevent extravasations. The dose injected was 0.2 mCi kg−1 body weight F18-FDG After the injection, patients waited for 60-90 min to allow FDG to penetrate the tissues and the PET/CT was then performed.

### Positron-Emission Tomography/Computed Tomography Scanning

PET/CT scanning was performed using a Siemens Biograph 6 True Point HD LSO integrated device. The first CT scan (130-136 keV; 60-90 mAs) was taken between the vertex-upper femur, in direct proportion to the weight of the patient, followed by a PET scan (performed in the same parameter range) for 3 min per bed. Additional imaging of the lower extremities was carried out in patients who had multiple metastases. The PET data obtained were processed with iterative reconstruction, and were converted into PET images, both with no attenuation correction made and with attenuation correction based on CT.

### Examination of the Images

All images obtained were examined on an LCD monitor as both attenuation-corrected and uncorrected multiplanar PET, CT, and FDG-PET/CT fusion cross-sections (maximum intensity projection=MIP), using the eSOFT software (Siemens).

### Assessment of Positron-Emission Tomography Computed Tomography Images

The lesions revealed in PET/CT scans were first evaluated visually. Each focal uptake identified in PET images was searched for in the corresponding CT cross sections. FDG uptakes that corresponded to the salivary gland, muscle, fatty tissue, and normal lymphoid tissue in CT cross-sections were accepted as physiological uptake. On the other hand, focal FDG uptakes corresponding to areas of abnormal soft tissue mass or lymph nodes in CT images were accepted as significant in terms of metastasis.

The point of concern that displayed the most intensive FDG uptake in the primary tumor region was identified and the SUVmax, which is a semi-quantitative measure of FDG uptake, was determined. A SUVmax value greater than 2.5 was used as a cut off for malignancy. The localization of the metastatic lymph node and the highest SUVmax of patients with lymph node metastasis were recorded along with the locations of distant metastasis, if present. Distant organ metastasis and distant lymph node metastasis were identified as distant metastasis (except loco-regional lymph nodes).

### Statistical Analysis

The normality of the data distribution was assessed with Shapiro-Wilk and one- sample Kolmogorov-Smirnov tests. Parametric data were presented as means ± standard deviation, and non-parametric data as median and minimum-maximum values. Nominal and categorical variables were presented as frequencies and percentages. The parametric distribution was compared with the Student t-test in independent groups, and with the Mann-Whitney U test in the rest. Survival was evaluated by the Kaplan-Meier method. Categorical variables were evaluated with chi-square and Fisher’s exact contingency tests. The Cox multivariate regression model was used to evaluate risk factors, which included gender, age, smoking, primary tumor SUVmax, metastatic lymph node, and stage that could impact survival. The tests were two-sided. P<0.05 was accepted as significant. Statistical analysis was performed with SPSS statistical software, version 17.0 (SPSS Inc, Chicago, Illinois, USA).

## RESULTS

**Figure 1:**
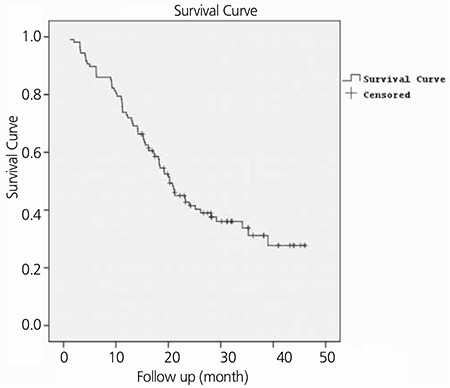
Kaplan Meier survival analysis of all patients. Patients were monitored for a 2-49 (20.2±2.07)-months follow-up period

In this retrospective study, we examined data from 112 patients who underwent FDG PET/CT for staging purposes. The histopathological diagnosis of two patients was leiomyoma. Endoscopic biopsy revealed in situ squamous cell carcinoma in three patients, and no pathologic FDG uptake was identified on their PET/CT scans. The follow-up visits indicated that these patients were disease-free. Within the remaining 107 patients, 48 (44.9%) were female and 59 (55.1%) were male. The age range of patients was 28-85 (56.6±12.3) years. After FDG PET/CT imaging, the patients were monitored for a 2-49 (20.2±2.07) month follow-up period (Figure 1). Most patients had squamous cell carcinoma on histopathology evaluation (Table 1).

Our results showed that esophageal cancer occurred most frequently in the lower thoracic esophagus (45.8%), and 3.7% in the upper thoracic esophagus. In the staging evaluation, only three patients had stage 1 disease, and they were combined with the group of patients with stage 2 disease. Thus, the three staging groups consisted of stages 1-2, 3, and 4 (Table 1).

The SUVmax value of the primary tumor was 2–48 in all groups, with a mean of 19.3±8.8. The highest SUVmax of metastatic lymph nodes were 2.9-60, with a mean of 10.4±9.1. Seventy patients had lymph node metastases. Lymph node metastases were classified as cervical, thoracic or abdominal, according to their localization. Metastases were detected most frequently in the thoracic area (43%), and least frequently in the cervical area (20.6%). Distant metastasis was present in 31.8% of the patients. Most of these metastases were located in the distant lymph nodes (18.7%) or adrenal gland (2.8%).

There was no significant correlation between tumor localization and survival, tumor localization and primary tumor SUVmax, or tumor localization and metastatic lymph node SUVmax (p=0.584, p=0.642, and p=0.632, respectively). Tumor localization was a neutral factor with respect to survival.

There was no significant association between the histopathology of the tumor and the primary tumor or metastatic lymph node SUVmax. The histopathology of the tumor did not appear to have an effect on survival (Table 2).

**Figure 2:**
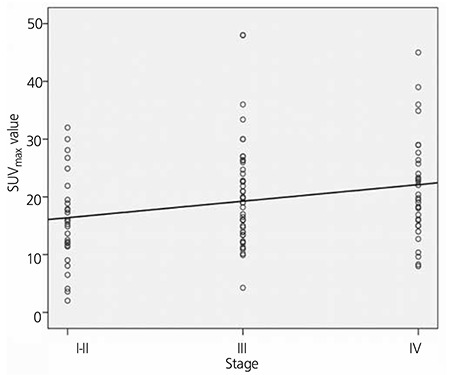
Correlation between tumor stage and SUVmax of the primary tumor. The mean SUVmax of the primary tumor was 15.5±7.6 for stages 1-2, 20.3±9.1 for stage 3, and 21.3±8.7 for stage 4. There was a significant difference between stage 4 patients and stages 1-2 patients (p=0.025)

Figure 2 shows the correlation between tumor stage and SUVmax of the primary tumor. As the tumor stage increased, the SUVmax of the primary tumor increased linearly. This increase was more significant in stage 4 patients than in stages 1-2 patients (with p=0.025). The SUVmax of the primary tumor was 15.5±7.6 for stages 1-2, 20.3±9.1 for stage 3, and 21.3±8.7 for stage 4.

The mean SUVmax of the primary tumor was higher in patients who had died, although not statistically significant (p=0.262).

Fifty percent of the 20 surviving patients had metastatic lymph nodes. Lymph node metastasis was detected in 75% of the 51 patients who died (p=0.003). The metastatic lymph node SUVmax were higher in patients who died as compared to those who survived [9 (3.1-60) vs. 5.1 (2.9-25.1), p=0.014]. When patients were grouped according to lymph node SUVmax value, patients with higher lymph node SUVmax had poorer outcomes. Furthermore, according to the ROC curve analysis, we identified that lymph node SUVmax value of 14.2 had a 80.8% sensitivity and 90% specificity for survival rate (p=0.017).

In terms of the correlation between distant metastasis and survival, four of the surviving patients (10%) had distant metastasis. Distant metastasis was also evident in 30 of the patients who died (45%), and its effect on survival was determined to be significant (p=0.001). Our results indicated thatlymph node metastases and distant metastases to the liver were related to survival (p=0.005 and p=0.04, respectively).

**Figure 3:**
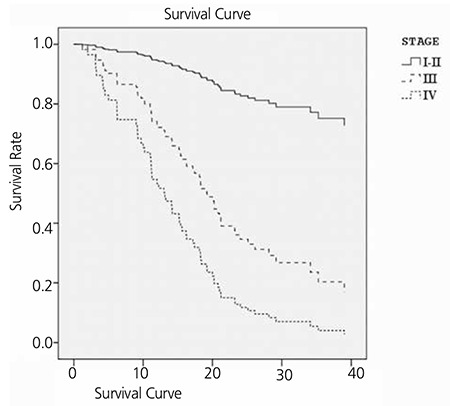
Kaplan-Meier curves show patient survival rates according to clinical stage. The median survival was 31.1 months for stage 1-2, 18.2 months for stage 3 and 15.1 for stage 4 (p=0.001)

Cox univariate and multivariate regression analyses were performed for the factors that could impact survival, which included gender, age, smoking, primary tumor SUVmax, metastatic lymph node SUVmax, the number of metastatic lymph nodes and tumor stage. Lymph node metastasis, lymph node SUVmax, and stage were determined to be significant in univariate analyses (p=0.04, p=0.014, and p=0.001, respectively). However, multivariate analysis determined that disease stage was the only independent variable associated with survival, which was notably significant in stages 3 and 4 (p=0.001). Compared with stage 1-2, stage 3 was associated with a 4.6-fold greater risk, and stage 4, an 8.2-fold greater risk mortality. The chi-square test indicated that the correlation between survival and cancer stage was significant (p=0.001) (Table 3) (Figure 3).

## DISCUSSION

Our findings suggest that the SUVmax of the primary tumor as determined by FDG PET/CT imaging that is used for staging patients with esophageal cancer is not predictive of survival. Our results indicated that the staging system currently used in clinical practice is effective regarding the survival of patients with esophageal cancer. Additionally, the lymph node SUVmax of patients with lymph node and distant metastasis had predictive value with regard to survival.

Esophageal cancer is aggressive and progresses rapidly in early stages via metastasis; thus, the long-term survival rate is low despite appropriate treatment (10). A review of the literature indicates a 5-year survival rate of 12% (11). Hong et al. (12) showed that patients followed-up for 5-25 months survived for a mean of 17.3 months. In another study, the mean survival was reported as 15.7 months over a 24-52 -month follow- up period (6). In our study, patients followed-up for 2-49 months after FDG PET/CT staging survived for 20.2 months, in line with previous findings.

In many studies, squamous cell-type esophageal cancer was related to poor prognosis (5,13); however, several studies reported that histopathologic type was not related to prognosis (4,6,14,15). In these studies, high SUVmax were attributed to increased Glut-1 expression (5,13). In our study, although the SUVmax were generally high, we observed that there was no difference according to histopathologic types. Moreover, histopathologic type was not associated with survival in our study; however, the squamous cell carcinoma subtype was over-represented in our patients.

Assessment of survival in advance plays an important role in the treatment of the disease (16). Classical staging has been shown to be associated with survival, and tumor tissue should be excised either surgically or by other methods, such as endoscopic.

In clinical practice, there is a need for less invasive and more accessible techniques to determine prognosis. The most frequently used approach is FDG PET/CT imaging. FDG PET/CT is a noninvasive imaging method used to quantify tumor metabolism and guide pre-treatment staging by identifying distant metastasis (10). The SUVmax in FDG PET/CT imaging has predictive value for survival, particularly in head-neck and lung cancer patients (17,18,19). In light of this information, many recent studies have cited SUVmax as an effective means of predicting survival in esophageal cancer patients (1,2,3,4,5,6,7,8,9).

Fukunaga et al. (4) and Kato et al. (5) determined that a high SUVmax value in the primary tumor was more effective in determining survival of patients with esophageal cancer as compared to low SUVmax. However, the most important deficiency of these two studies was that multivariate analysis was not performed, making the significance of their findings uncertain. In a study of patients with esophageal carcinoma, the primary tumor SUVmax was associated with survival (9); however, most of the patients were in early stages of the disease that was assessed clinically and pathologically.

In a study of 47 patients by Hong et al., (12) the SUVmax was not related to survival; only the quantity of abnormal uptake on FDG PET/CT imaging appeared to have an association. We should note that in our study clinical staging was not included in survival analysis, and that there were fewer early-stage than advanced-stage patients. A study conducted by van Westreenen et al. (20) reported that high SUVmax was related with poor prognosis but not survival in patients with esophageal carcinoma. Choi et al. (2) showed that tumor volume and presence of lymph nodes displaying FDG uptake were independently associated with survival while SUVmax was not. In a study of 40 patients with distal esophageal cancer, the SUVmax of the primary tumor was not related to survival; this outcome was attributed to the histopathology, which only included adenocarcinoma (21). In all these studies, the SUVmax was determined to predict survival by univariate analysis, with the exception of the following two studies; using univariate analysis of survival could be the most important deficiency in these studies. Two large, multi-centered, randomized, prospective studies reported that the SUVmax of the primary tumor was not associated with survival (8,22). However, as these two studies only involved patients suitable for curative treatment, the debate on this issue continues. A recent study indicated that tumor volume, tumor length and total lesion glycolysis were significant prognostic factors for overall survival, but SUVmax was not (23). We did not evaluate PET/CT parameters such as tumor volume and total lesion glycolysis in the present study. However, our data indicated no correlation between the SUVmax of the primary tumor and survival, according to both univariate and multivariate analyses. The higher number of patients with advanced-stage disease as compared to early-stage patients in our study, and histopathologically, the fewer adenocarcinomas as compared to squamous cell carcinomas may have prevented identification of a correlation between survival and the SUVmax of the primary tumor. In those studies showing a relationship between SUVmax and survival, the SUVmax varied between 3 and 12 (4,6,7). The much higher mean SUVmax in our study was attributed to the high rate of squamous cell carcinoma and the high proportion of advanced-stage patients.

Another important finding of our study is that the presence of metastatic lymph node and distant metastasis are predictive of survival. The high SUVmax of the metastatic lymph nodes were negatively associated with survival. We found that lymph node SUVmax had high sensitivity and specificity for survival rate. A review of previous studies indicated that many factors have been investigated in terms of prediction of survival in patients with esophageal cancer; however, the correlation between the SUVmax of metastatic lymph nodes and survival is reported herein for the first time.

Cheze-Le Rest et al. (6) determined that while the existence of two or more local lymph nodes with FDG uptake affected survival, distant organ and/or lymph node uptake did not. When the methodology of this study was reviewed, it is seen that distant organ and/or lymph node metastasis was not stated separately; uptake in more than one field in FDG PET imaging was considered as distant metastasis. Many studies have reported that lymph node uptake is an independent predictive factor for survival (2,3,6,7).

In our study, consistent with these previous reports, lymph node uptake was predictive of survival as indicated by univariate analysis. Additionally, distant organ metastasis was independently negatively associated with survival. Distant organ metastasis, particularly those to the liver, had the greatest influence on survival, consistent with previous reports (24).

In many studies, pathological and clinical staging was independently associated with survival (2,3,8). However, several others have reported that clinical staging was not associated with survival (6,25). In the latter two studies, in which no relationship between staging and survival was determined, FDG PET was not used as the quantitative method and conventional methods were used for staging. In our study, consistent with the literature, pathological and clinical staging had an independent predictive value for survival in patients with advanced-stage disease. Furthermore, the SUVmax of the primary tumor was high in advanced stages of the disease, and this elevation was significant, particularly in stages 1-2 and stage 4.

### Limitations

The retrospective nature of this study may be the most important limitation. The heterogeneity of the patient group, in terms of stage and histopathology, represents another limitation. Additionally, because most patients did not undergo surgery, pathological staging of these patients could not be assessed. It is well known that the false positivity and false negativity rates of lymph node SUVmax value are high in detecting lymph node metastasis, due to accompanying inflammatory disease and microscopic metastasis (26). In other words, lymph node SUVmax does not necessarily indicate a true lymph node metastasis, without confirmation by pathological evaluation. Nevertheless, in the present study, pathological assessment of lymph nodes was not performed. There were two reasons for this; first, most patients were treated without surgery due to advanced stage disease with distant organ metastasis, and second the retrospective design of the study did not allow performing additional pathological evaluation. Another limitation of the present study was lack of follow-up of treatment regimens and treatment center of the patients. We also did not evaluate PET/CT parameters such as metabolic tumor volume and total lesion glycolysis.

## CONCLUSIONS

According to our findings, the SUVmax of the primary tumor did not have a predictive value in terms of survival in patients with esophageal cancer. Consistent with previous studies, the predictive value of staging by FDG PET/CT imaging in determining survival of esophageal cancers was found to be reliable, making this method feasible for clinical practice. Although the negative effect of lymph node metastasis on survival of patients with esophageal cancer is well known, the negative effect of high lymph node SUVmax on survival is a new parameter, which should be considered for clinical practice.

At least two professional editors, both native speakers of English, have checked the English in this document. For a certificate, please see: http://www.textcheck.com/certificate/zfj1td.

## Figures and Tables

**Table 1:**
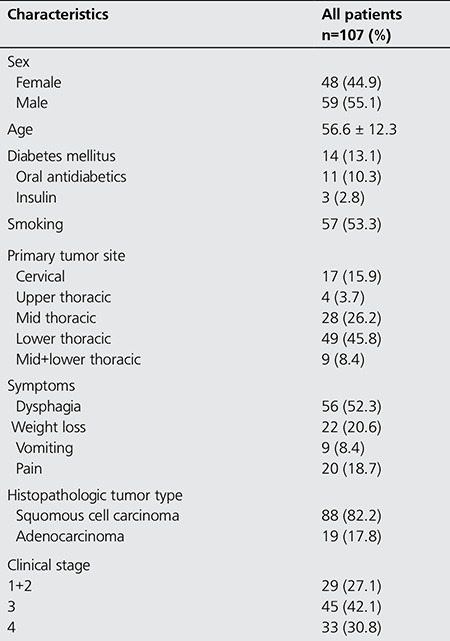
Patient characteristics

**Table 2:**
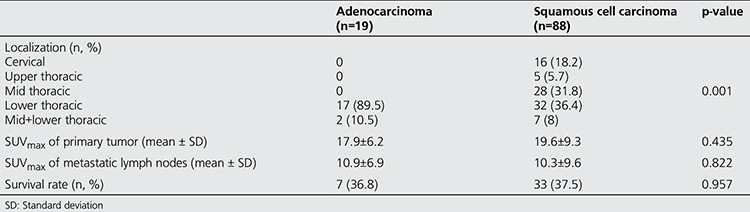
Correlation between localization, histopathological type, SUVmax and survival rate

**Table 3:**
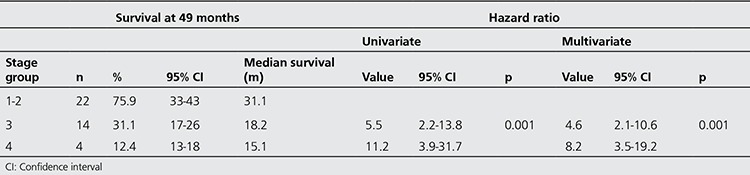
Univariate and Cox-multivariate regression analysis of the correlation between clinical stages and survival rate
